# Regulatory T cells: the 2025 Nobel prize in physiology or medicine

**DOI:** 10.48101/ujms.v131.13770

**Published:** 2026-01-16

**Authors:** Kailash Singh

**Affiliations:** Medical Cell Biology, Uppsala University, Uppsala, Sweden

The 2025 Nobel Prize in Physiology or Medicine has been awarded to Mary Brunkow, Fred Ramsdell, and Shimon Sakaguchi *‘for their discoveries concerning peripheral immune tolerance*’. The award honours the discovery of a unique subset of immune cells – Regulatory T cells (Tregs) – which differ fundamentally from most immune cells that typically promote inflammation. In contrast, Tregs suppress immune activation, maintain immune homeostasis, and prevent immune-mediated tissue destruction. Without Tregs, the immune system fails to regulate itself, leading to catastrophic multi-organ autoimmunity.

**Figure:**
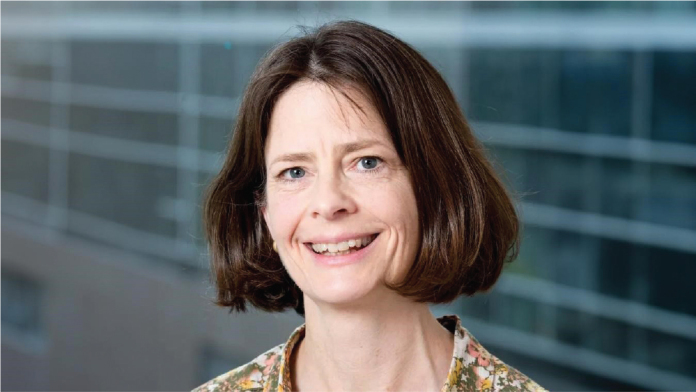
Mary E. Brunkow, Institute for Systems Biology, Seattle, WA, USA

**Figure:**
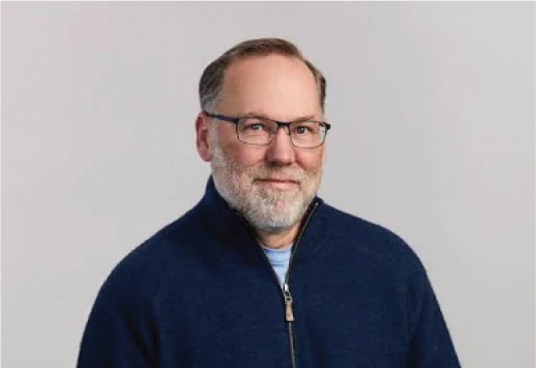
Fred Ramsdell, Sonoma Biotherapeutics, USA

Although Tregs are now well-established, their discovery followed a long and challenging path. In the 1970s, the concept of ‘suppressor T cells’ was proposed and linked to the I-J locus, which was initially believed to encode a key regulatory molecule, present in Major Histocompatibility Complex (MHC) a part of H-2 complex (1). But in 1983, the I-J locus was shown not to exist in suppressive T cells, casting doubt on the entire suppressor T-cell field (2). Between 1982 and 1990, research teams including Shimon Sakaguchi, Don Mason, and Fiona Powrie continued to investigate T-cell subsets with suppressive activity in rodent models. Their work identified T-cell populations capable of preventing autoimmune disease and wasting syndromes in mice and rats (3, 4), keeping the field alive despite the skepticism of many researchers.

**Figure:**
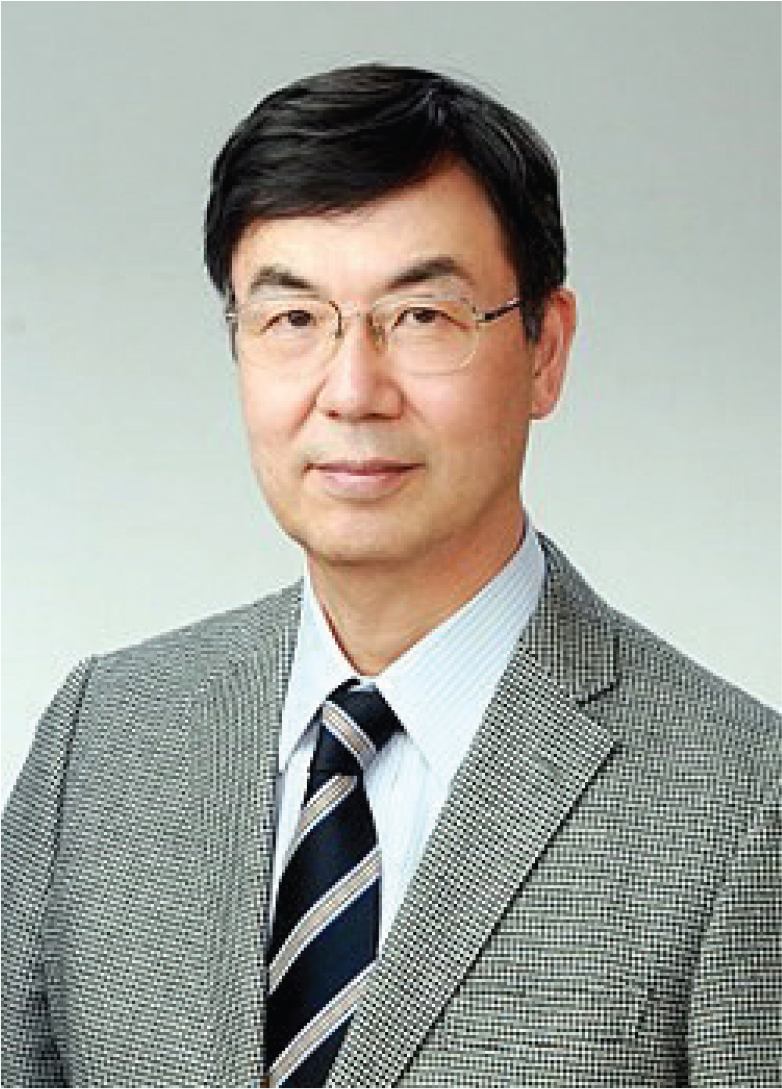
Shimon Sakaguchi, Institute for Frontier Medical Sciences, Kyoto University, Kyoto, Japan, Immunology Frontier Research Center (IFReC), World Premier International Research Center (WPI), Osaka University

A major breakthrough came in 1995, when Sakaguchi’s group identified CD25, the α-chain of the IL-2 receptor, as a candidate marker distinguishing Tregs from conventional T cells (5).

In parallel – but from a genetics perspective rather than immunology – Brunkow and Ramsdell discovered that a mutation in the mouse Foxp3 gene (the *scurfy* mutation) caused the lethal autoimmune disorder later termed IPEX (immune dysregulation, polyendocrinopathy, enteropathy, X-linked syndrome). This discovery rapidly led to the identification of FOXP3 mutations in human IPEX patients, firmly establishing FOXP3 as a central regulator of immune tolerance (6–8).

The role of FOXP3 in Tregs became clear in 2003, when three independent groups led by Sakaguchi, Ramsdell, and Alexander Rudensky demonstrated that FOXP3 is the master transcription factor that specifies Treg identity. Forced expression of FOXP3 converted conventional T cells into Tregs, revealing how this transcription factor rewires self-reactive T cells during thymic development – shifting their response from inflammatory to suppressive programs (9–11).

The discovery of FOXP3 ignited an explosion of research into Treg biology and their contribution to immune regulation in health and disease. However, challenges remain: FOXP3 is intracellular, complicating Treg isolation for translational work, and Treg plasticity under inflammatory conditions remains a subject of active investigation.

This year’s Nobel Prize recognizes not only the profound clinical implications of Tregs but also the decades of rigorous, methodical science that made their discovery possible. While the Prize honours Brunkow, Ramsdell, and Sakaguchi, it also acknowledges the many research teams whose combined efforts shaped this transformative field. Importantly, it highlights the powerful synergy between human genetics and mouse immunology – and stands as an endorsement of persistent, careful science that ultimately rebuilt a once-discredited hypothesis into a pillar of modern immunology.
